# A Case of COVID-19 and Methicillin-Resistant Staphylococcus aureus (MRSA) Coinfection Resulting in MRSA Empyema

**DOI:** 10.7759/cureus.59254

**Published:** 2024-04-29

**Authors:** Hironori Kobayashi, Shunta Takeuchi, Tadasuke Ikenouchi, Nozomi Goto, Masahiro Ogawa

**Affiliations:** 1 Respiratory Medicine, Handa City Hospital, Handa, JPN

**Keywords:** surgery, drainage, empyema, coinfection, coronavirus disease 2019, mrsa

## Abstract

Bacterial coinfections in patients with COVID-19 are rare; however, coinfection with *Staphylococcus (S.) aureus* is relatively common. No detailed report of patients with COVID-19 and methicillin-resistant *S. aureus* (MRSA) coinfection has been documented. Herein, we present a case of a patient with COVID-19 and MRSA coinfection who developed MRSA empyema after pneumonia and bacteremia. A 59-year-old man was admitted to the intensive care unit for treatment of COVID-19 and bacterial pneumonia with septic shock. He was initially treated with antibiotics, antiviral agents, and steroids. On the third day of admission, MRSA was detected in both sputum and blood cultures. Although he was treated with appropriate vancomycin doses with monitoring of renal function and serum vancomycin concentrations, he developed bilateral pleural effusions one week after starting treatment. Initially, the bilateral pleural effusions were thought to have been caused by hypoalbuminemia. However, bilateral chest drainage was performed due to the onset of left-sided chest pain. The left-sided pleural effusion was exudative, whereas the right-sided pleural effusion was transudative. MRSA was later detected on culture of the left-sided effusion but not the right-sided effusion. Based on the findings of the pleural fluid examination, the patient was diagnosed with left-sided empyema. His symptoms and radiographic findings improved after a repeat drainage of the left pleural effusion. Vancomycin was administered for 28 days, and the patient was discharged on the twenty-eighth day of admission. These findings highlight the importance of pleural fluid examination for the prompt diagnosis of pleural infection. Early diagnosis of empyema and prompt chest drainage may help avoid the need for surgery. This report could contribute to the clinical management of patients with COVID-19 and MRSA coinfection.

## Introduction

Coronavirus disease 2019 (COVID-19) is a highly infectious disease caused by severe acute respiratory syndrome coronavirus 2 [1]. Although bacterial coinfection among patients with COVID-19 is low, the rate of *Staphylococcus (S.) aureus* coinfection is relatively high [2,3]. In Japan, bacterial coinfection at the time of COVID-19 diagnosis is rare (55/1,863, 3.0%), and only two cases (0.11%) of COVID-19 and methicillin-resistant *S. aureus* (MRSA) coinfection have been reported [3]. The clinical characteristics of COVID-19 with MRSA coinfection remain unclear. Although MRSA empyema has been reported following COVID-19 [4,5], to our knowledge, no cases of concurrent MRSA empyema and COVID-19 have been reported to date.

In this article, we present a case of COVID-19 and MRSA coinfection in a patient who developed MRSA empyema after pneumonia and bacteremia.

## Case presentation

**Figure 1:**
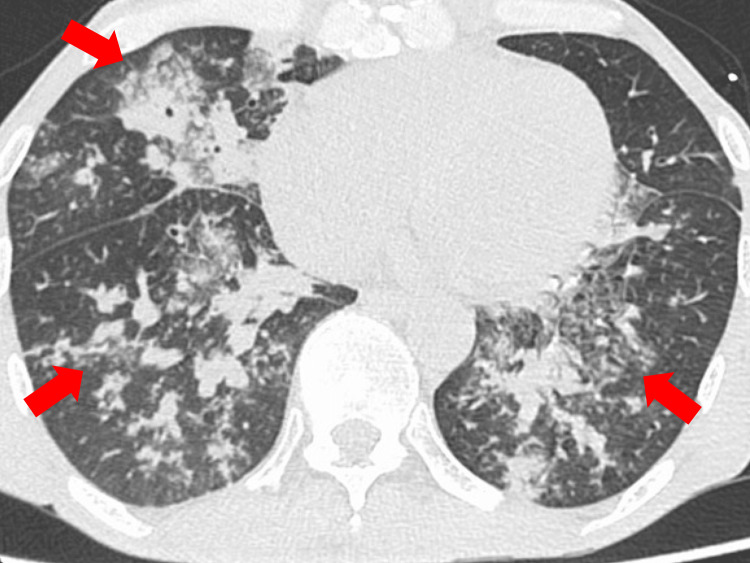
Chest computed tomography imaging on the day of admission Chest computed tomography showing infiltrating shadows in both lungs, consistent with bacterial pneumonia

A 59-year-old man presented to the emergency department with a two-day history of dyspnea. On arrival at the emergency department, he was in septic shock and required fluid resuscitation and vasopressors. His family had COVID-19; therefore, he underwent a SARS-CoV-2 antigen test, which was positive, and was diagnosed with COVID-19. His medical history included surgery for pancreatic cancer, performed 11 months previously, and gastroesophageal reflux disease, which had been treated with esomeprazole and pancrelipase. He had last been hospitalized nine months previously. Chest computed tomography (CT) revealed infiltrating shadows in both lungs, suggestive of bacterial pneumonia (Figure 1). No cavitary lesion was observed on chest CT. Sputum Gram stain revealed a relatively good-quality specimen with Geckler classification group 4 and phagocytosis of clusters of Gram-positive cocci. He was admitted to the intensive care unit for treatment of COVID-19, bacterial pneumonia, and septic shock.

Ceftriaxone, azithromycin, vancomycin, remdesivir, and steroids were administered as initial treatment. Further, high-flow nasal cannula oxygen therapy was administered for respiratory distress, and noradrenaline was administered for shock.

On the third day of admission, MRSA was detected in both the sputum and blood cultures. Therefore, he was diagnosed with MRSA pneumonia and bacteremia with COVID-19 coinfection, and ceftriaxone was discontinued. Blood cultures retested on the fourth day of admission were negative. Further, transthoracic echocardiography did not reveal any vegetation of the heart valves.

**Figure 2:**
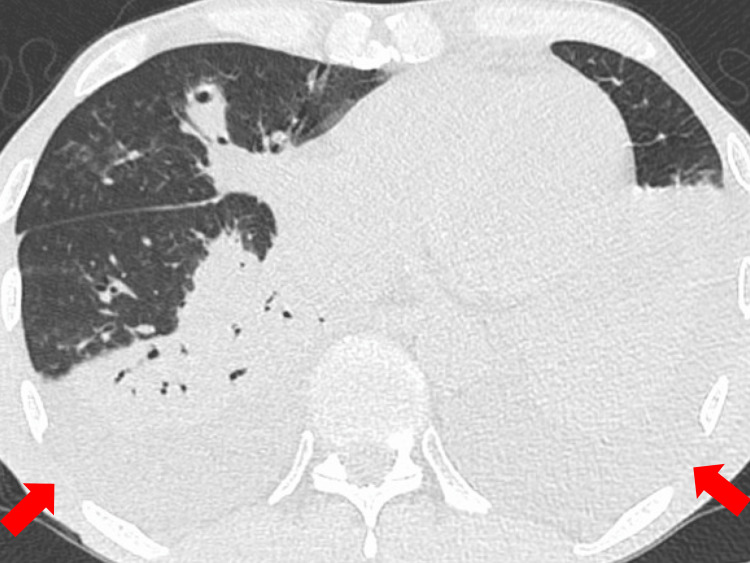
Chest computed tomography imaging on the eighth day of admission Chest computed tomography showing bilateral pleural effusions, despite a reduction in the infiltrating shadows

Gradually, his respiratory and cardiovascular status improved; therefore, he was discharged from the intensive care unit to the ward on the fourth day of admission. Although the patient was treated with an appropriate dose of vancomycin, with monitoring of renal function and serum vancomycin concentrations, high-grade fever reappeared on the sixth day of admission. On the eighth day of admission, chest CT revealed bilateral pleural effusions despite improvement in the infiltrating shadows (Figure 2). On the ninth day of admission, left chest drainage was performed due to the onset of left chest pain and suspected pleuritis. The pleural fluid was yellow and turbid. Pleural fluid analysis showed a nucleated cell count of 8,245/µL with 86.5% neutrophils; levels of lactate dehydrogenase, protein, and glucose of 449 U/L, 2.4 g/dL, and 181 mg/dL, respectively; and a pH of 7.2. MRSA was subsequently identified on pleural fluid culture, and the patient was diagnosed with MRSA empyema. Additionally, right chest drainage was performed with drainage of yellow-colored clear pleural fluid. Pleural fluid analysis showed a nucleated cell count of 204/µL with 28.3% neutrophils; levels of lactate dehydrogenase, protein, and glucose of 88 U/L, 1.9 g/dL, and 157 mg/dL, respectively; and a pH of 7.6. No pathogens were detected on the pleural fluid culture of the right-sided effusion.

The patient had a VIRSTA score of 4, suggestive of infective endocarditis [6]. Therefore, transesophageal echocardiography was performed on the thirteenth day of admission; however, this did not reveal any vegetation.

**Figure 3:**
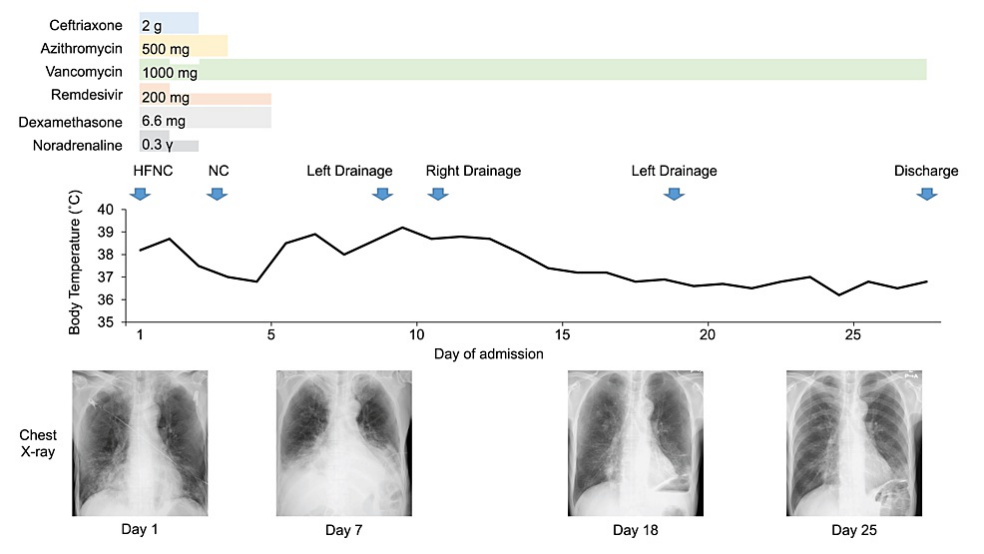
Clinical course of this case The patient received ceftriaxone (2 days), azithromycin (3 days), vancomycin (28 days), remdesivir (5 days), and dexamethasone (5 days). He also received high-flow nasal cannula oxygen therapy due to respiratory failure and noradrenaline (2 days) due to septic shock. His respiratory and cardiovascular status gradually improved following treatment. In addition, bilateral chest drainage was performed due to recurrent high fever, chest pain, and bilateral pleural effusions. His symptoms and radiographic findings improved after a repeat drainage of his left pleural cavity. He was discharged on the twenty-eighth day after admission. HFNC, high-flow nasal cannula; NC, nasal cannula

On the thirteenth day of admission, the chest tube was removed because the effusion had almost completely stopped draining. However, residual pleural effusion was observed; therefore, the left chest drainage was repeated at a different site on the nineteenth day of admission. Subsequent radiography confirmed that the pleural effusion had almost disappeared, and the chest tube was removed on day 21. Vancomycin was discontinued after 28 days, and the patient was discharged on the twenty-eighth day of admission. The clinical course of the patient is summarized in Figure 3.

## Discussion

The patient had COVID-19 and MRSA coinfection with pneumonia and bacteremia at the time of admission, and subsequently developed MRSA empyema. Early diagnosis of empyema and prompt chest drainage helped avoid the need for surgery. This case has two important findings as follows: (1) COVID-19 and MRSA can coinfect and empyema can occur as a complication of MRSA pneumonia, and (2) prompt chest drainage and pleural fluid examination can help avoid surgery in patients with MRSA empyema.

This patient presented coinfection with COVID-19 and MRSA and developed MRSA empyema after one week. Vancomycin was administered to treat the MRSA infection in accordance with our hospital’s antibiogram and epidemiology [7]. Only MRSA was detected in both sputum and blood cultures on the third day of admission. Based on the CT, sputum Gram stain, and culture findings, the patient was considered to have developed MRSA bacteremia as a complication of MRSA pneumonia. The patient was treated with appropriate vancomycin doses according to his degree of renal function, which was confirmed by monitoring his serum vancomycin concentration. However, he developed bilateral pleural effusions one week after starting vancomycin treatment. Bilateral chest drainage revealed left-sided MRSA empyema, confirmed through pleural fluid culture, whereas the right-sided pleural effusion was transudative according to Light’s criteria [8]. The patient’s MRSA bacteremia did not persist, and although his VIRSTA score was suggestive of infective endocarditis, transesophageal echocardiography did not reveal any vegetation. Based on these findings, MRSA was believed to have invaded the thoracic cavity directly from the left lung, leading to empyema.

In addition, prompt chest drainage helped avoid surgery in this patient. Guidelines on managing pleural empyema recommend early drainage of the infected material from the pleural space and suggest that surgical referral should be considered within a few days of chest tube insertion in cases of ongoing sepsis, radiological persistence, and/or clinical deterioration [9,10]. In this patient, bilateral chest drainage was performed because of recurrent high fever and left chest pain. Based on the subsequent pleural fluid examination, the patient was diagnosed with left-sided empyema. When the patient’s symptoms did not resolve following the first chest drainage, the left chest drainage was repeated and his symptoms and radiographic findings improved. In symptomatic patients with pleural effusions, pleural fluid examination is important to enable early diagnosis of pleural infection. Early diagnosis of empyema and prompt chest drainage can help avoid surgery, as was observed in the present case.

## Conclusions

This case report describes a case of COVID-19 and MRSA coinfection, complicated by MRSA empyema due to direct infiltration of MRSA from the left lung to the thoracic cavity. The patient was treated with vancomycin for 28 days, with appropriate monitoring of blood levels, and underwent recurrent chest drainage and pleural fluid examination, guided by his clinical response. He recovered without requiring surgery. In patients with MRSA empyema, appropriate antibiotic therapy and prompt chest drainage can help avoid surgery. This illustrative case could contribute to the clinical management of patients with COVID-19 and MRSA coinfection.
